# Supplementary material for: Effects of a Sodium Phosphate Electrolyte Additive on Elevated Temperature Performance of Spinel Lithium Manganese Oxide Cathodes
Source: Materials (Basel). 2021 Aug 19;14(16):4670. doi: 10.3390/ma14164670 (PMC8402163; doi:10.3390/ma14164670)
Supplement: Supplementary file 1 [file materials-14-04670-s001.zip › materials-1334415-supplementary.pdf]

materials

Supplementary Materials

Effects of a Sodium Phosphate Electrolyte Additive on Elevated Temperature Performance of Spinel Lithium Manganese Oxide Cathodes

Minsang Jo [1], Seong-Hyo Park [1] and Hochun Lee [1,2,*]

[1] Department of Energy Science and Engineering, Daegu Gyeongbuk Institute of Science and Technology (DGIST), Daegu 42988, Korea; alstkdwh@dgist.ac.kr (M.J.); serafpsh@dgist.ac.kr (S.-H.P.)
[2] Energy Science and Engineering Research Center, Daegu Gyeongbuk Institute of Science and Technology (DGIST), Daegu 42988, Korea
* Correspondence: dukelee@dgist.ac.kr

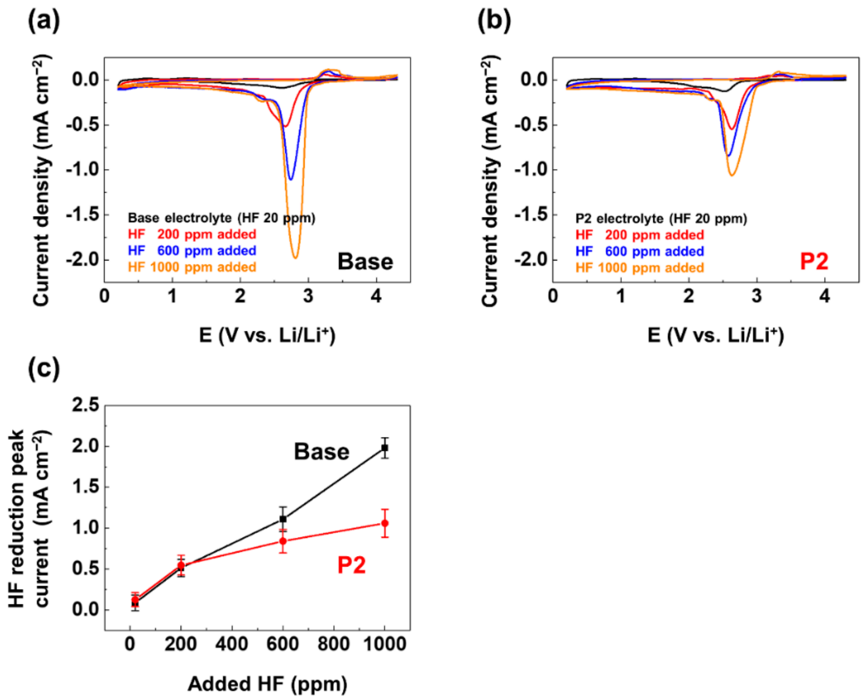

Figure S1. Cyclic voltammograms of the (**a**) base and (**b**) P2 electrolytes. The working electrode was Pt and the scan rate was 10 mV s^{-1}. (**c**) HF reduction peak current in the base and P2 electrolytes. Reproduced with permission [1]. Copyright 2020, The Electrochemical Society.

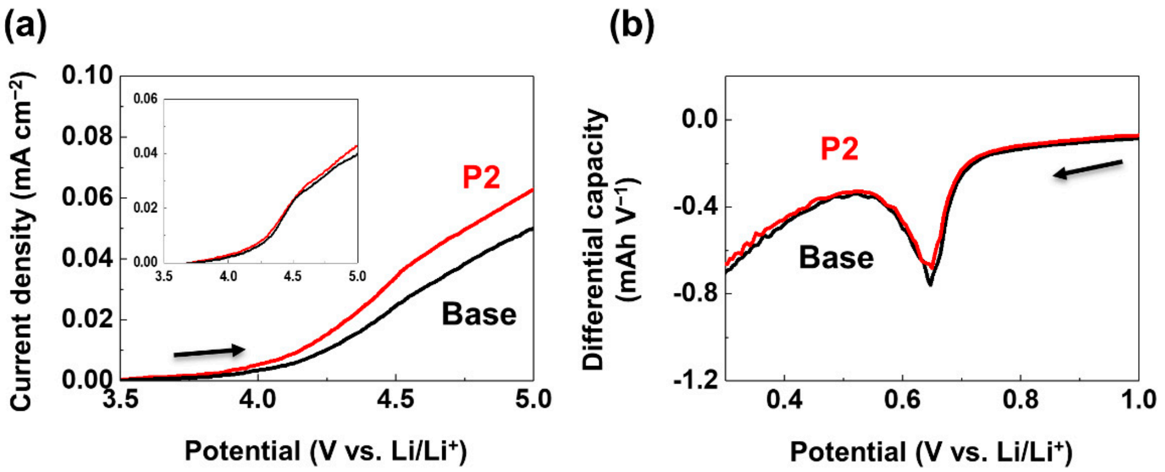

Figure S2. (**a**) Linear sweep voltammograms of the base and P2 electrolytes. Working electrode was Pt and the scan rate was 1 mV s^{-1}. The second scan is shown in the inset. (**b**) Differential capacity versus potential (dQ/dV vs. V) curves of graphite/Li cells with the base and P2 electrolytes during the first lithiation process. Arrows indicate the scan direction. Reproduced with permission [1]. Copyright 2020, The Electrochemical Society.

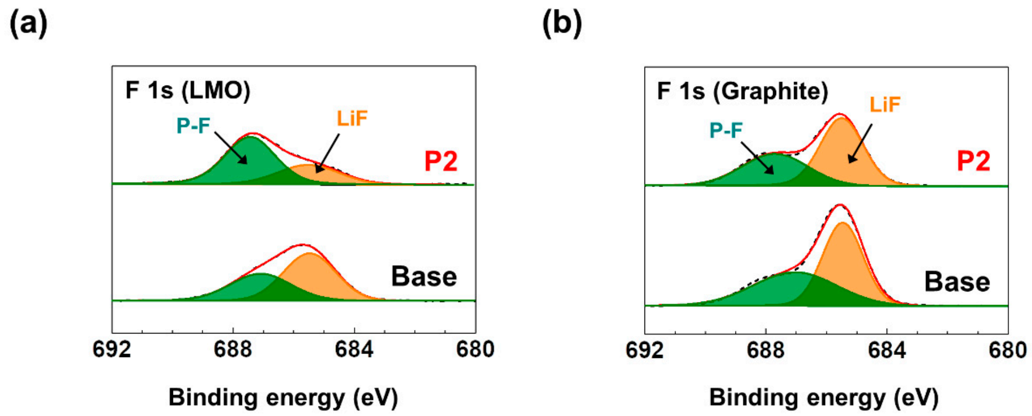

Figure S3. F 1s XPS spectra of (**a**) LMO cathodes and (**b**) graphite anodes after three cycles at 25 °C. Dotted lines denote the experimental spectra and solid lines denote the best-fitted results.

Table S1. Composition of CEI layer on the LMO cathodes obtained from TEM/EDX analysis (wt%).

Sample	Mn	O	F	P	Na
Cycled in the base	58.23	35.85	5.44	0.48	0.00
Cycled in the P2	55.19	38.21	4.63	0.68	1.29

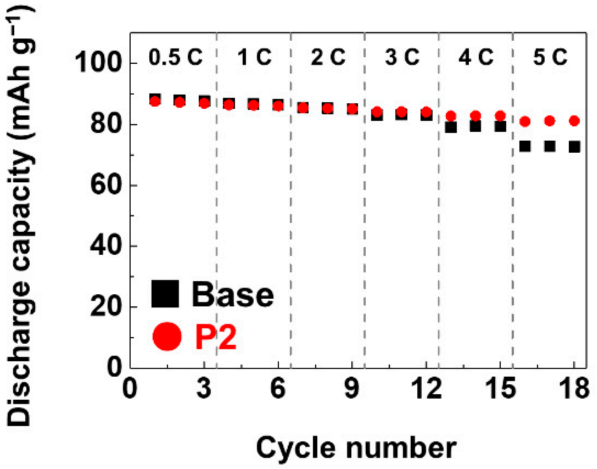

Figure S4. Discharge rate capability of LMO/graphite cells at 25 °C.

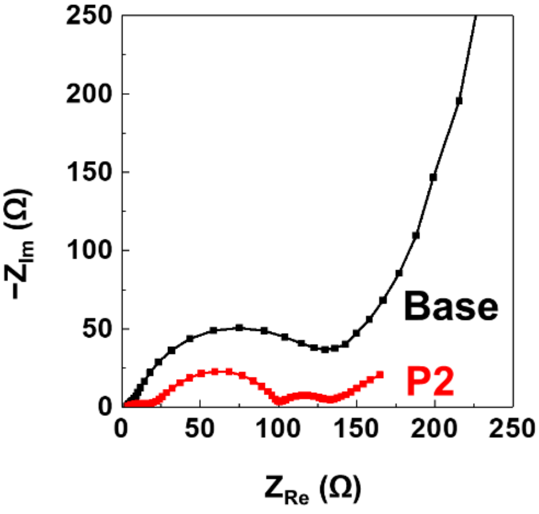

Figure S5. Nyquist plots of LMO/graphite cells with and without P2 additive. The cells were cycled three times and set to SOC 100 before the EIS measurements.

References

1. Jo, M.; Park, S.-H.; Lee, H. NaH_2PO_4 as an Electrolyte Additive for Enhanced Thermal Stability of $LiNi_{0.8}Co_{0.1}Mn_{0.1}O_2$/Graphite Batteries. *J. Electrochem. Soc.* **2020**, *167*, 130502.
